# Impact of Social, Economic, and Healthcare Factors on the Regional Structure of Antibiotic Consumption in Primary Care in Poland (2013–2017)

**DOI:** 10.3389/fpubh.2021.680975

**Published:** 2021-07-29

**Authors:** Anna Olczak-Pieńkowska, Waleria Hryniewicz

**Affiliations:** Department of Epidemiology and Clinical Microbiology, National Medicines Institute, Warsaw, Poland

**Keywords:** antibiotic consumption, antibiotic resistance, antibiotic stewardship, primary care, antibiotic consumption factors

## Abstract

Antibiotic resistance is one of the most important public health threats worldwide. Antimicrobial misuse and overuse are well-recognized risk factors for the resistance emergence and spread. Monitoring of antibiotic consumption (AC) is an important element in strategies to combat antibiotic resistance. As a result of AC surveillance in Poland, regional differences in AC levels were observed. This study aimed to characterize the regional AC in the period 2013–2017 in primary care in Poland and to assess the possible determinants that influence the AC at the regional level. The study analyzed relationships between AC and its factors (grouped in three different categories: demographic, economic, and related to the organization of healthcare). Data covered AC in primary care in 5 years period (2013–2017) from all 16 Polish voivodeships. The AC primary care data were sales data, expressed in defined daily doses (DDD) according to the WHO methodology. The yearly data on demographic, economic, and related healthcare factors were downloaded from the Central Statistical Office of Poland. Standard statistical parameters were used to characterize the AC. Non-standardized regression coefficients were used to estimate the quantitative dependence of variables. The strongest correlation was demonstrated with factors related to employment, female reproductive activity, mobility of the population, the number of outpatient consultations, and the number of dentists. A correlation was also found between population mobility and density. Recognized risk factors for increased AC should be a priority for interventions implementing and disseminating rational antibiotic policy.

## Introduction

Antibiotic resistance is one of the leading public health problems worldwide. It has become an everyday concern for medical personnel, scientists, and institutions creating health policy (1, 2). It is also increasingly becoming an everyday concern for patients and their families struggling with ineffective antibiotic therapies. It poses a considerable risk to health and life, generates enormous costs, and constitutes a great organizational challenge for healthcare. Antimicrobial misuse and overuse are well-recognized risk factors for resistance emergence and spread (3, 4). Therefore, monitoring of antibiotic consumption (AC) is one of the main tools to plan actions focused on improving antimicrobial usage and an essential element of strategies to combat antibiotic resistance (1, 2).

Poland belongs to a group of 10 European countries with the highest community level of AC. It has remained above the average in Europe through the years 1998–2018. In 2018, Poland was in fifth place among the EU countries with the highest AC. The average level of community AC (23 DDD per 1,000 inhabitants per day, DID) was over 2.5 times higher than in the Netherlands (8.9 DID), which was the country with the lowest AC (5).

In a previous study, we observed that despite the regional similarity in AC structure and trends, there were significant regional differences in AC levels in Poland (6). Thus, we decided to characterize the regional AC in 2013–2017 in primary care in Poland and to assess possible determinants (demographic, economic, or healthcare-related) that influence the AC at the regional level. To indicate the factors that may reduce the high level of AC in Poland is of great importance for effective actions aimed at rational antibiotic therapy and, thus, indirectly for an effective strategy to combat antibiotic resistance. For example, countries like Sweden or France show that such activities are possible if these activities are continuous and systematic.

## Materials and Methods

The study analyzed the relationship between data on AC and its factors grouped in three different categories: demographic, economic, and related to healthcare (Supplementary Table 1). All variables were collected for the period 2013–2017 from all 16 Polish voivodeships and total country data. The AC primary care data were from sales data (pharmacy level). They were provided through the courtesy of IQVIA, a company monitoring the pharmaceutical market. The supplied data on the numbers and contents of packages were used in calculations of consumption to express AC in defined daily doses (DDD) according to the methodology proposed by the WHO Collaborating Centre for Drug Statistics Methodology (7). The consumption was analyzed at the level of the entire group of antibacterial for systemic use, J01 in the Anatomical Therapeutic Chemical (ATC) classification system, specific J01 subgroups: tetracyclines (J01A), penicillins (J01C), cephalosporins, and other beta-lactams (J01D), sulfonamides and trimethoprim (J01E), macrolides, lincosamides, and streptogramins (J01F), aminoglycosides (J01G), quinolones (J01M), other antibacterials (J01X) and specific antibacterials: doxycycline (J01AA02), amoxicillin (J01CA04), phenoxymethylpenicillin (J01CE02), amoxicillin-clavulanic acid (J01CR02), cephalexin (J01DB01), cefadroxil (J01DB05), cefuroxime (J01DC02), erythromycin (J01FA01), clarithromycin (J01FA09), ciprofloxacin (J01MA02), and nitrofurantoin derivate (J01XE01). For all substances, we considered only the oral administration route (parenteral was negligible in primary care). The nomenclature of the subgroups of antibiotics was used in accordance with the ATC classification; however, consumption of some subgroups was represented by a single substance, e.g., J01E was represented only by sulfamethoxazole with trimethoprim and J01F only by clarithromycin, azithromycin, and clindamycin (Table 1).

**Table 1:** Antimicrobials used in the period 2013–2017 in primary care in Poland by ATC names [substances of consumption over 0.1 defined daily doses (DDD) per 1,000 inhabitants per day (DID) are listed].

**Subgroups ATC code**	**Substance (name)^**a**^**	**Substance** **(ATC code)** ^**a**^	**Consumption in Defined Daily Doses per 1,000 inhabitants per year**				
			**2013**	**2014**	**2015**	**2016**	**2017**
J01A	Doxycycline	J01AA02	2.13	1.86	1.95	1.91	1.92
J01C	Amoxicillin	J01CA04	5.35	4.84	3.77	5.24	5.15
	Phenoxymethylpenicillin	J01CE02	0.16	0.21	0.33	0.31	0.30
	Amoxicillin-clavulanic acid	J01CR02	3.85	3.74	3.60	4.38	4.21
J01D	Cefuroxime	J01DC02	2.16	2.08	2.25	2.45	2.62
	Cefaclor	J01DC04	0.13	0.10	0.08	0.10	0.13
J01E	Sulfamethoxazole and trimethoprim	J01EE01	0.24	0.47	0.58	0.47	0.39
J01F	Clarithromycin	J01FA09	1.60	1.43	1.18	1.53	1.62
	Azithromycin	J01FA10	1.23	1.25	1.54	1.65	1.85
	Clindamycin	J01FF01	0.69	0.72	0.80	0.79	0.77
J01M	Ciprofloxacin	J01MA02	0.71	0.71	0.79	0.77	0.77
	Norfloxacin	J01MA06	0.31	0.30	0.31	0.29	0.28
	Levofloxacin	J01MA12	0.13	0.15	0.19	0.24	0.29
J01X	Nitrofurantoin derivatives	J01XE01	3.18	3.49	3.81	2.54	4.03
P01A	Metronidazole	P01AB01	0.19	0.16	0.17	0.17	0.19

*ATC, anatomical therapeutic chemical.*

a *All listed antibiotics were for the oral route of administration; parenteral use was negligible in primary care in Poland*.

The English names of voivodeships were used according to Eurostat nomenclature (8).

The yearly data on demographic, economic, and healthcare factors was downloaded from the Central Statistical Office of Poland (Główny Urzad Statystyczny—GUS) (9).

The following measures and estimates were used: consumption expressed in DDD and DID; mean (average) values of AC; range and SD; coefficient of variation; rankings; coefficient of determination; and linear regression.

Non-standardized regression coefficients were used to estimate the quantitative dependence of variables. The r-squared values (*R*^2^) were used to estimate what percentage of the variation of the variable was explained by the model. *P*-values ≤ 0.05 were considered statistically significant. The results presented and listed in Table 2 display only relationships that showed correlation with statistical significance.

**Table 2:** Factors influencing antimicrobial consumption in the period 2013–2017 in Poland (only factors found to influence consumption are listed, all factors analyzed are listed in the Supplementary Table 1).

	**Factor**	**Antibacterial (name)**	**Antibacterial (ATC code)**	**Beta**	***R*^**2**^**	***p*-value**	**Statistical significance**
1.	Unemployed for more than a year (% of the population by the duration of unemployment: over 1 year)	Antimicrobials for systemic use	J01	−0.176	0.585	0.002	*p* < 0.05
2.	Total fertility rate	Tetracyclines	J01A	−1.015	0.615	0.00004	*p* < 0.05
		Penicillins	J01C	−4.172	0.643	0.00141	*p* < 0.05
		Nitrofurantoin derivate	J01XE01	1.408	0.480	0.0245	*p* < 0.05
3.	Females per 100 males	Clarithromycin	J01FA09	0.139	0.849	3.34E-07	*p* < 0.05
4.	Population density per 1 square km	Tetracyclines	J01A	0.002	0.615	2.003E-06	*p* < 0.05
5.	National and international migration for permanent residence per 1,000 population	Amoxicillin-clavulanic acid	J01CR02	0.516	0.808	3.88E-15	*p* < 0.05
6.	Dentists per 10,000 inhabitants	Penicillins	J01C	0.281	0.381	0.019	*p* < 0.05
		Amoxicillin-clavulanic acid	J01CR02	0.176	0.538	0.016	*p* < 0.05
		Cefuroxime	J01DC02	0.109	0.572	0.028	*p* < 0.05
		Clindamycin	J01FF01	0.026	0.669	0.018	*p* < 0.05
7.	Number of medical consultation provided in primary care per capita	Penicillins	J01C	0.872	0.643	0.025	*p* < 0.05
8.	Average monthly net income per capita in households	Other antibacterials	J01X	0.001	0.394	0.001	*p* < 0.05

*ATC, anatomical therapeutic chemical*.

## Results

The average AC in the period 2013–2017 in primary care in Poland was 24.10 DID and fluctuated in the range of 23.45–25.73 DID. The average AC in the following years was 23.50 DID (2013), 22.48 DID (2014), 25.61 DID (2015), 23.43 DID (2016), and 25.52 DID (2017). The lowest AC level was 18.17 DID in 2014 in Warminsko-Mazurskie, the highest was 30.58 DID in 2017 in Swietokrzyskie voivodeship. During the analyzed years, the average AC at the voivodship level increased by 2.02 DID. A decrease in AC was recorded only in four voivodships (Lubuskie by 1.66, Podkarpackie by 1.62, Pomorskie by 0.36, and Podlaskie by 0.16 DID). The most significant increase in AC was recorded in Swietokrzyskie (by 5.66 DID), followed by Lubelskie (by 4.32 DID) and Warminsko-Mazurskie (by 4.17 DID).

**Figure 1:**
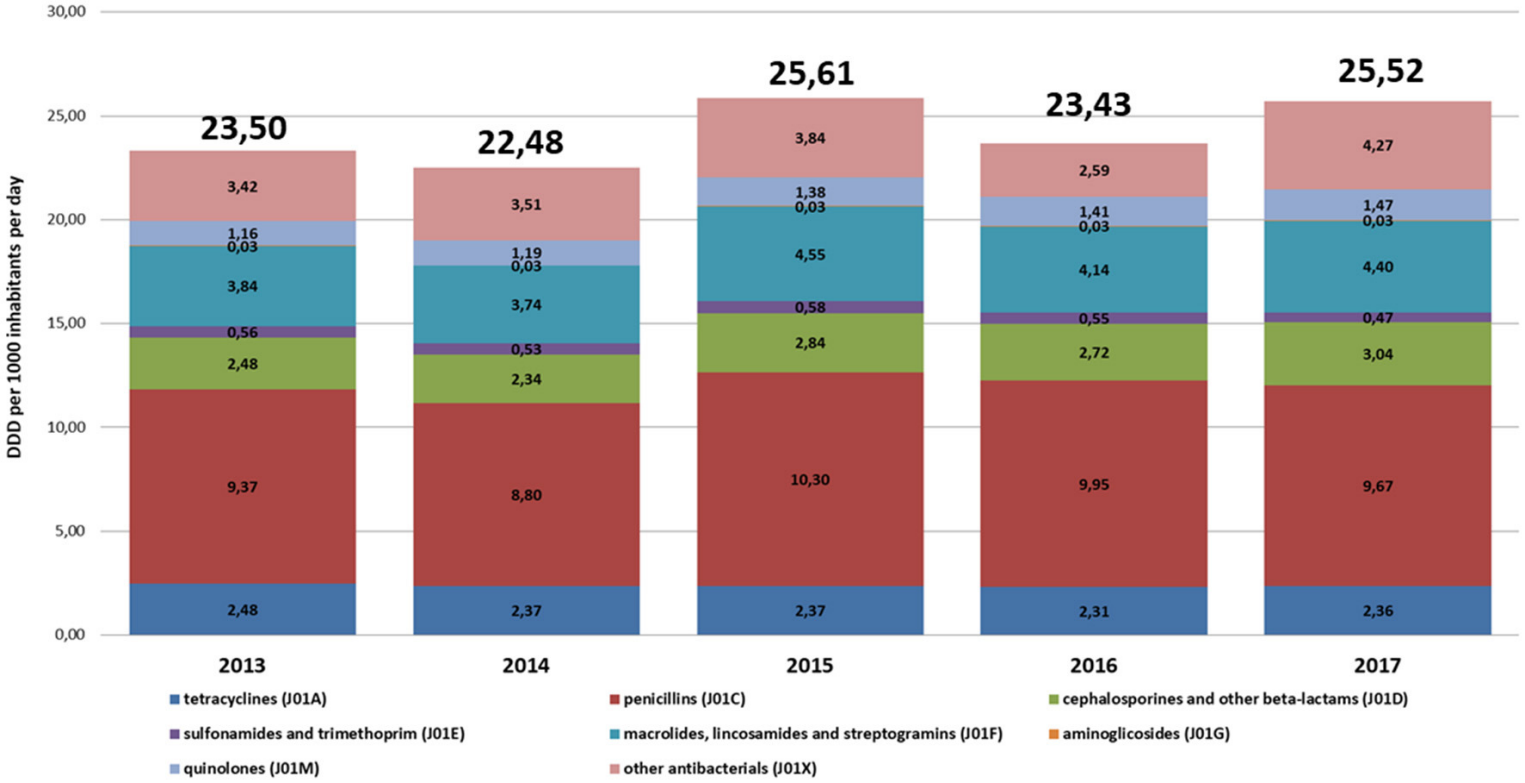
The structure of consumption of antibacterials for systemic use (J01) by subgroups in primary care in Poland (2013–2017, defined daily doses, DDD, per 1,000 inhabitants per day, DID).

**Figure 2:**
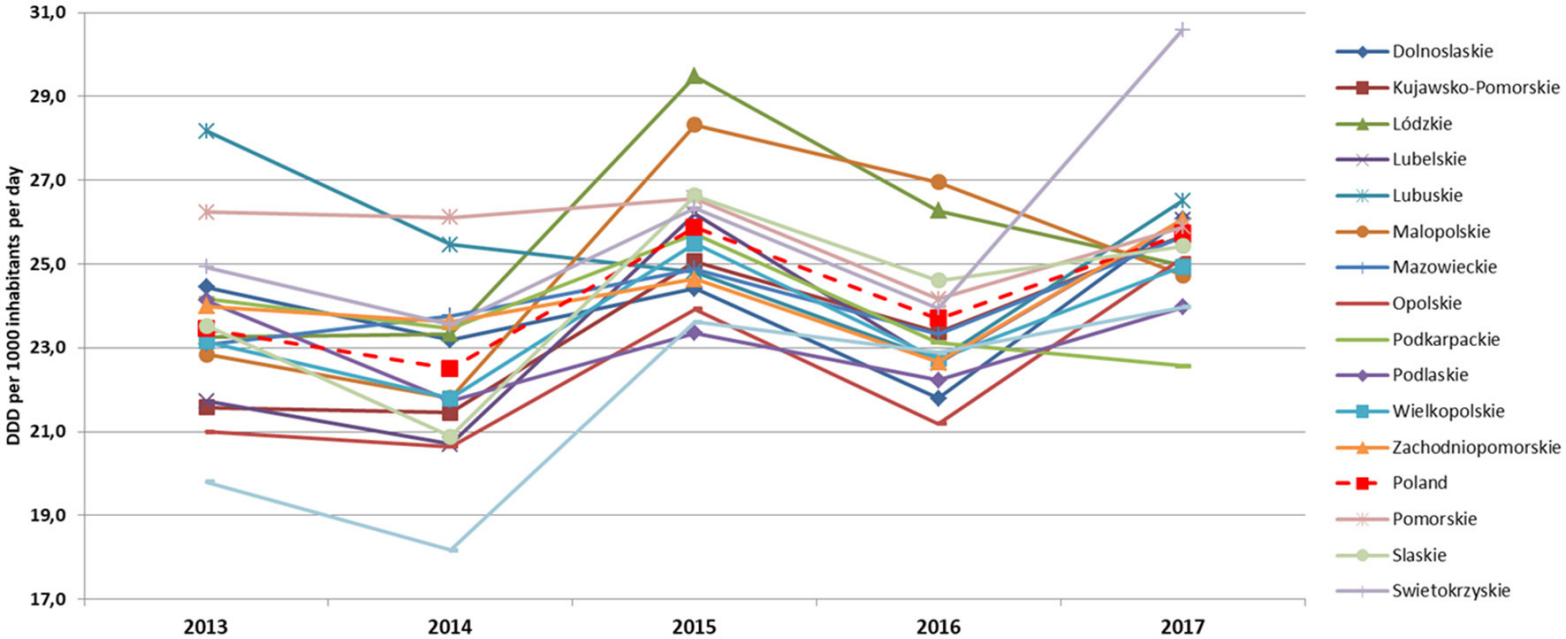
Antimicrobial consumption trends in the period 2013–2017 in primary care in Polish voivodships (DDD and DID).

Although the consumption levels differed between voivodeships, overall trends and structure of most frequently consumed antibiotics were similar in the analyzed years (Figures 1, 2).

The most commonly used subgroups of antibiotics were the following (in decreasing order): penicillins (J01C), macrolides, lincosamides and streptogramins (J01F), other antibiotics (J01X), tetracyclines (J01A), cephalosporins, monobactams, and carbapenems (J01D), quinolones (J01M), and sulfonamides with trimethoprim (J01E). Voivodships with the lowest consumption levels of J01 in individual years were as follows: Warminsko-Mazurskie (2013 and 2014), Podlaskie (2015), Opolskie (2016), and Podkarpackie (2017). The highest consumption level of J01 were in as follows: Lubuskie (2012 and 2013), Pomorskie (2014), Lódzkie (2015), Malopolskie (2016) and Swietokrzyskie (2017).

In this study, we found a correlation between J01 consumption and the percentage of the people who were unemployed for more than a year. If the unemployment rate measured by this indicator was higher by 10%, it resulted in J01 consumption lower by 1.761 DID (*R*^2^ = 0.342). Lubuskie Voivodeship was an example of where the unemployment indicator was low during the analyzed period, while J01 consumption was one of the highest. On the other hand, in Podlaskie and Mazowieckie Voivodeships, the J01 consumption was low compared with other voivodeships, while the unemployment indicator was relatively high.

The total fertility rate (TFR) showed a correlation with the consumption of tetracyclines (J01A) and penicillins (J01C). In voivodeships, where the average number of children born to a woman over her lifetime was higher by one child, the J01A consumption was lower by 1.015 DID (*R*^2^ = 0.615). The TFR higher by one correlated with J01C consumption lower by 4.171 DID (*R*^2^ = 0.643). An example of the voivodeship, where the J0A and J01C consumption remained relatively high, while TFR low, was Swietokrzyskie.

Total fertility rate was also correlated with nitrofurantoin derivative (J01XE01) consumption. In voivodeships with the TFR higher by one, consumption of J01XE01 was higher by 1.408 DID (*R*^2^ = 0.480). The examples were Mazowieckie and Pomorskie, where we observed the highest J01XE01 consumption and the highest TFR.

Voivodeships with 10 more females per 100 males were correlated with oral clarithromycin consumption higher by 1.387 DID (*R*^2^ = 0.849).

Considering the population density, voivodeships, where the number of inhabitants was higher by 10 people per 1 square km, showed that the consumption of J01A was higher by 0.02 DID (*R*^2^ = 0.615).

Consumption of oral amoxicillin-clavulanic acid (one of the most frequently prescribed antimicrobial in primary care in Poland) showed a correlation with the national and international migration for permanent residence per 1,000 population. Voivodeships, with this indicator higher by 10, showed that the consumption of amoxicillin-clavulanic acid was higher by 5.162 DID (*R*^2^ = 0.808).

Antibiotic consumption also showed a correlation with the number of dentists per 10,000 inhabitants. No such correlation was found for the number of doctors and nurses. In voivodeships with the number of dentists higher by 10 per 10,000 inhabitants, consumption of J01C was higher by 2.807 DID (*R*^2^ = 0.381), amoxicillin-clavulanic acid (J01CR02) higher by 1.764 DID (*R*^2^ = 0.538), cefuroxime higher by 1.091 DID (*R*^2^ = 0.572), and metronidazole higher by 0.257 DID (*R*^2^ = 0.669). The examples of the voivodeships with the relatively high J01CR02 consumption and a high number of dentists per 10,000 inhabitants were Malopolskie and Lubuskie. In Opolskie, we observed the lowest J01CR02 consumption and the lowest number of dentists per 10,000 inhabitants. An example of the opposite correlation was Slaskie.

The consumption of J01C was correlated with the number of medical consultations provided in primary care per capita. Voivodeships with 10 more medical consultations given per capita showed about 8.716 DID higher consumption of J01C (*R*^2^ = 0.643).

Consumption of the J0X group showed a correlation with the average monthly net income per capita in households; however, the correlation seemed weak (*R*^2^ = 0.394). Monthly net income per capita higher by 1,000 PLN correlated with higher J01X consumption by 0.823 DID.

## Discussion

High AC in Poland is conditioned by several specific factors. The Polish generic medicines market is one of the biggest in Europe. The share of generic drugs accounts for more than 50%, with growing trends. Therefore, prices of medicines in Poland, including antibiotics, are among the lowest in Europe and low enough to be affordable by the majority of the population (10, 11). In addition, almost everybody in Poland has health insurance covering antibacterial drug reimbursements, making the final cost to the consumer even lower.

As shown in an earlier study (6), seasonal trends in AC were noted, with a peak in winter and early spring, when outbreaks of influenza and other seasonal respiratory viral infections prevail. In this study, in addition to the seasonal trends, we noted significant variability in AC between regions (voivodships) of Poland. The strongest correlations were demonstrated with factors related to employment, female reproductive activity, mobility of the population, the number of outpatient consultations, and the number of dentists.

Less frequent use of antibiotics among the unemployed, observed in our study, may be conditioned by their limited financial resources and limited access to healthcare. The unemployed in Poland are covered only by basic health insurance on the condition that they register at the employment office; however, unlike employees, they do not receive sickness benefits when they are ill. On the other hand, higher AC in voivodeships with “higher professional activity” (lower percentage of the unemployed) can indicate the greater tendency to use antibiotics in the case of infection, under pressure to return to work faster. A similar result was noted in a Eurobarometer report indicating that the unemployed are least likely to take antibiotics and least likely to have obtained antibiotics under medical supervision (12).

In addition to factors related to the professional activity of the population, we also found an interesting correlation between J01C and J01A consumption and fertility rate. It is visible that women in the childbearing group were more careful about taking penicillins (of which major part is represented by amoxicillin-clavulanic acid and amoxicillin) and tetracyclines (mainly doxycycline). This observation reflects that only a few antibiotics are allowed to be used during the pregnancy and breastfeeding periods due to their toxicity to the fetus (13). At the same time, in the group of women of the childbearing age, despite lower consumption of J01A and J01C, high consumption of furazidine, a derivative of nitrofuran, was observed (nitrofurantoin is not available in Poland). This is an antibiotic used to treat uncomplicated urinary tract infections (UTI), available in Poland over-the-counter (OTC) and is strongly advertised. In the analyzed years, the average consumption of furazidine was about 3.41 DID (rising from 3.18 DID in 2013 to 4.03 DID in 2017), which comprised about 15% of the consumption of the entire J01 group. These observations suggest that young women, who are more prone to UTI, may strongly misuse and overuse furazidine. The obvious reason for this is its OTC availability and aggressive advertisement for UTI. This issue is of great concern because of the documented resistance profiles of UTI pathogens. Surveillance studies published in 2001 and 2008 showed that the nitrofurantoin resistance of *Escherichia coli* (the leading cause of UTI) in Poland was already at the highest level in Europe before OTC availability of the drug in Poland: 8.8 and 4.4% in 2001 and 2008, respectively (14, 15); however, according to the recent (2013) Polish data, nitrofurantoin resistance of *E. coli* was at 32.8% and may be a consequence of the increasing consumption of the OTC furazidine, often without medical indications (16). This is a dangerous trend, particularly among women of childbearing age, and requires urgent interventions.

A systemic review and meta-analysis published in 2016 showed that women were prescribed antibiotics more often than men, and macrolides were the class with the highest gender differences (more frequent use by women). The authors of the meta-analysis consider macrolides as a group of drugs mainly used for the treatment of respiratory tract infections (RTI) (17). One of the recent Dutch studies also showed a higher incidence of respiratory symptoms in women than men (18). Observation indicates high clarithromycin consumption by the female gender. In Poland, clarithromycin is one of the most often prescribed drugs of the macrolide group. It is recommended for the treatment of community RTI, in combination therapy to eradicate *Helicobacter pylori*, and as prophylaxis after pertussis exposure (19). This antibiotic is cheap and is available in various formulations with friendly posology, and all this may contribute to its over-prescription (20). The gender differences in consumption might be explained by gender differences in the incidence of the infection. Unfortunately, no Polish data is available on this subject, and several other studies have shown contradictory results (21–23). The abovementioned meta-analysis from 2016 indicated the higher UTI prevalence in women; however, it showed equal gender consumption of quinolones as the group is widely used to treat UTI (17). Male patients are more likely to be diagnosed with less frequent but complicated UTI, which may determine antibiotic choice and use.

The important “demographic” factors associated with AC, in this study, was population density per square km correlated with tetracyclines and national and international migration for permanent residence per 1,000 population correlated with amoxicillin-clavulanic acid. The higher concentration and more intensive population movements intensify the contacts between people and can facilitate the spread of pathogens and most probably infections, resulting in higher antibiotics use. More frequent prescription in higher density populations may also reflect prescribing antibiotics “just in case” at seasonal peaks of RTI.

Lower infection incidence (especially the RTI) in lower population density regions was documented (24, 25). The role of reduced social contacts in limiting the spread of RTI can also be easily observed in the current development of the COVID-19 pandemic.

Several studies showed a correlation of AC with the percentage of the elderly in a population. Research studies in Polish confirmed the link between AC and age of the patients, showing that AC variation was strictly linked with the age of the patients (26). A similar connection was established by Swedish authors who mentioned children aged 0–5 years and patients ≥ 75 years of age among socio-demographic factors connected with high AC (27). These results did not show a statistically significant relationship between the age of the population and AC; however, when looking at particular voivodships, we observed the highest levels of AC in the voivodeships with the highest percentage of the elderly, while in the voivodeships with the lowest AC, the percentage of elderly was also the lowest. The correlation between AC and age might have physiological and social conditions. In general, the immune system of the elderly is impaired (immunosenescence) comparing with younger age groups (28). It leads to higher infection risk and incidence, resulting in more frequent prescription of antibiotics. This risk in elderly patients is further increased by the higher incidence of comorbidities by their recurrent contact with healthcare and long-term facilities residency (29).

We also observed a connection between the AC and the numbers of medical personnel per 10,000 inhabitants, looking at factors related to healthcare. The correlation was found only for dentists but not for doctors and nurses. This might suggest that the availability of doctors did not condition prescribing frequency, and their decisions were motivated by other independent factors (e.g., recommendations, morbidity, etc.). Consumption of the whole group of penicillins (J01C) and specific antibiotics, such as amoxicillin-clavulanic acid, cefuroxime, and clindamycin, were correlated with the number of dentists per 10,000 inhabitants. Dental care in Poland is dominated by the private sector, which is easily accessible than the public one. Professional training for dentists mostly concerns the improvement of dental techniques, often organized by commercial companies, and there is no mandatory training on antibiotic therapy. The over-prescribing and improper prescribing of antibiotics by dentists were also concluded by other studies, e.g., Canadian and the worldwide review of studies investigating patterns of antibiotic use by this medical group. Authors described practices of dental patients being given antibiotics for a range of medical complaints, including “toothache” (30, 31). A recent study from Poland on the knowledge of antimicrobial therapy of dental students before their final exams (graduation) showed that more than 10% of dentists would prescribe antibiotics for viral infections, so more focus should be given to this topic (32).

Although no correlation was found in this study between the AC and the number of doctors, the number of medical consultations provided in primary care appears to influence AC. It means that the prescription of antibiotics is higher when visits of patients are intensified, though during seasonality peaks. Therefore, those periods are burdened with the risk of excessive use of antibiotics (6, 33) and should be the target of educational campaigns on rational antibiotic therapy.

There are several limitations in this study. The sales data do not necessarily reflect the actual consumption of the analyzed group of drugs; however, at the moment, sales data are the most reliable source of AC available, both at the national and regional levels. Although the determinants of AC are multifactorial, we were able to discern some areas, which may contribute to antibiotic misuse and overuse and which should be addressed in antibiotic policy planning. We analyzed, for the first time in Poland, the factors that may affect AC in primary care, and the results might be the basis for interventions focused on rational antibiotic therapy. The AC data should be monitored and analyzed continuously. Data on AC linked to information on diagnoses made as a basis for antibiotic prescription would also be valuable. Such data were not generated so far in Poland; however, e-prescriptions introduced in 2020 may facilitate such comparisons in the future.

## Conclusion

This study indicated the differences in the level of AC in different regions of Poland. Several determinants influence AC. The group at risk of inappropriate or unnecessary use of antibiotics are women (especially those of reproductive age). Employment is a factor that influences AC. Areas with increased population movements and densities should be a priority in planning and implementing educational measures for the rational use of antibiotics and infection control measures. OTC availability of furazidine on the Polish market allows the access of this antibiotic beyond medical control and may be the reason for its overuse in all Polish regions. Access to the OTC antibiotics is a matter of concern, and it requires reconsideration with respect to the national antibiotic strategy. Educational activities and recommendations in the field of antibiotic therapy should be promptly disseminated and implemented among dentists. A more detailed analysis of the actual indications and diagnoses that led to the prescription of antimicrobials could result in a reduction in AC, which remains high in primary care in Poland. Knowledge about the AC should be considered by stakeholders as the basis for antibiotic policy improvement.

## Data Availability Statement

The raw data supporting the conclusions of this article will be made available by the authors, without undue reservation.

## Author Contributions

AO-P collected and analyzed the data. Both authors contributed to the design of the research, edited, and approved the final version of the manuscript.

## Conflict of Interest

The authors declare that the research was conducted in the absence of any commercial or financial relationships that could be construed as a potential conflict of interest.

## Publisher's Note

All claims expressed in this article are solely those of the authors and do not necessarily represent those of their affiliated organizations, or those of the publisher, the editors and the reviewers. Any product that may be evaluated in this article, or claim that may be made by its manufacturer, is not guaranteed or endorsed by the publisher.
