# Supplementary material for: Impact of Social, Economic, and Healthcare Factors on the Regional Structure of Antibiotic Consumption in Primary Care in Poland (2013–2017)
Source: Front Public Health. 2021 Jul 29;9:680975. doi: 10.3389/fpubh.2021.680975 (PMC8358207; doi:10.3389/fpubh.2021.680975)
Supplement: Supplementary file 1 [file Table_1.DOCX]

Supplementary Material

**Supplementary Table 1.** Index of all analyzed factors (demographic, economic and healthcare related and economic).

| **DEMOGRAPHIC FACTORS** | |
| --- | --- |
|  | Number of population |
|  | Total number of females |
|  | Total number of males |
|  | Females per 100 males |
|  | Population by median age |
|  | Females by median age |
|  | Males by median age |
|  | Number of residents in urban areas |
|  | Number of residents in rural areas |
|  | Percentege of residents of urban areas in total population |
|  | Natural increase per 1000 population |
|  | Total fertility rate |
|  | Employed population aged 15 and more by Labour Force Survey (LFS) |
|  | Employed per 1000 population |
|  | Unemployed registered (for the last day of the year) |
|  | Registered unemployment rate [%] |
|  | Unemployed as % of population by duration of unemployment: over 1 year |
|  | Unemployed as % of population by age: 24 years or less |
|  | Unemployed as % of population by age: 50 years or more |
|  | Population at pre-working age (for the last day of the year) |
|  | Population at working age (for the last day of the year) |
|  | Population at post-working age (for the last day of the year) |
|  | Population at non-mobility working age per 100 population at working age |
|  | Children below 5 years |
|  | Children below 18 years |
|  | Population below 25 years as % of total population |
|  | Population over 65 years as % of total population |
|  | Density of population (person/1km^2^) |
|  | Infant deaths per 1000 live births |
|  | National and international migration for permanent residence per 1000 population |
|  | Number of pre-primary education establishments |
|  | Number of nursery schools |
|  | Children attending pre-primary education establishments |
|  | Children attending nursery schools |
|  | Children attending nursery schools at age 6 years |
|  | Number of students per 10 thousand inhabitants |
|  | Doctors (active practice for the last day of the year) |
|  | Tourism accommodation – the number of accommodations in a given year |
| **HEALTHCARE RALETED FACTORS** | |
|  | Doctors (active practice for the last day of the year) |
|  | Dentists (active practice for the last day of the year) |
|  | Nurses (active practice for the last day of the year) |
|  | Pharmacists (active practice for the last day of the year) |
|  | Number of out-patient facilities in urban area |
|  | Number of out-patient facilities in rural area |
|  | Population per 1 hospital bed in general hospital |
|  | Population per 1 out-patient facility |
|  | Population per 1 pharmacy |
|  | Number of pharmacies generally accessible |
|  | Number of out-patient medical consultations given per 1 inhabitant |
|  | Number of dental consultations in primary care per 1 inhabitant |
|  | Influenza incidence per 100 thousand inhabitants |
|  | Tuberculosis incidence per 100 thousand inhabitants |
| **ECONOMIC FACTORS** | |
|  | Gross domestic product (in million PLN) |
|  | Gross domestic product per 1 inhabitant (PLN) |
|  | Average monthly per capita net receipts in households – net receipts (PLN) |
|  | Average monthly per capita net receipts in households – disposable income (PLN) |
|  | Average monthly per capita net receipts in households – available income (PLN) |
|  | Average monthly per capita net receipts in households – domestic retirement pensions (PLN) |
|  | Average monthly per capita net receipts in households – domestic disability pension (PLN) |
|  | Average monthly net income per 1 inhabitant in households – national disability pension (PLN) |
|  | Average monthly net income per 1 inhabitant in households – income from other social benefits (PLN) |
|  | Average monthly net income per 1 inhabitant in households – domestic unemployment benefits (PLN) |
|  | Average monthly net outgoings per 1 inhabitant in households – net outgoings (PLN) |
|  | Average monthly net outgoings per 1 inhabitant in households – expenditures (PLN) |
|  | Average monthly net outgoings per 1 inhabitant in households – consumer goods and services (PLN) |
|  | Average monthly net outgoings per 1 inhabitant in households – food and non-alcoholic beverages (PLN) |
|  | Average monthly net outgoings per 1 inhabitant in households – health (PLN) |
|  | Average monthly net outgoings per 1 inhabitant in households – recreation and culture (PLN) |
|  | Average monthly net outgoings per 1 inhabitant in households – education (PLN) |
